# Ongoing outbreaks of hepatitis A among men who have sex with men (MSM), Berlin, November 2016 to January 2017 – linked to other German cities and European countries

**DOI:** 10.2807/1560-7917.ES.2017.22.5.30457

**Published:** 2017-02-02

**Authors:** Dirk Werber, Kai Michaelis, Marius Hausner, Dagmar Sissolak, Jürgen Wenzel, Julia Bitzegeio, Anne Belting, Daniel Sagebiel, Mirko Faber

**Affiliations:** 1 State Office for Health and Social Affairs (LAGeSo), Berlin, Germany; 2 Robert Koch Institute, Berlin, Germany; 3 Local Public Health Authority, Berlin Mitte, Germany; 4 National Consultant Laboratory for Hepatitis A and Hepatitis E, Institute of Clinical Microbiology and Hygiene, University Medical Center Regensburg, Regensburg, Germany; 5 Bavarian Health and Food Safety Authority (LGL), Oberschleißheim, Germany

**Keywords:** Sexually transmitted infection, Hepatitis A, outbreak, men who have sex with men, molecular subtyping

## Abstract

Since 14 November 2016, 38 cases of hepatitis A have been notified in Berlin; of these, 37 were male and 30 reported to have sex with men (MSM). Median age of MSM cases is 31 years (range: 24–52 years). Phylogenetic analysis revealed three distinct sequences, linking cases in Berlin to those in other German cities and to clusters recognised in other European countries in 2016.

On 14 December 2016, the local public health authority (LPHA) of the Berlin district Mitte informed the State Office for Health and Social Affairs (SOHSA) in Berlin, of two male cases of hepatitis A, notified in calendar week 50, who identified themselves as men who have sex with men (MSM). At that time, no increase in hepatitis A cases was apparent in the notification data.

Immediately following this information, we enhanced epidemiological and virological surveillance of hepatitis A in Berlin and report here preliminary findings.

## Enhanced surveillance and molecular analyses

In the absence of an increase of hepatitis A in the notification data of Berlin in calendar week 50/2016, we (arbitrarily) considered a possible outbreak beginning as of calendar week 46/2016 (starting 14 November), i.e. four weeks (mean incubation period of hepatitis A) before the hepatitis A cases in MSM were first recognised. This coincided with when notified hepatitis A cases started to be predominantly male adults. We applied the case definition that is also used for surveillance purposes in Germany, i.e. symptomatic disease defined as fever or one of the following: abdominal discomfort, increase in serum transaminases, jaundice, plus laboratory confirmation, i.e. detection of hepatitis A virus (HAV) nucleic acid or HAV-specific IgM or a distinct increase in IgG [1]. We requested all 12 LPHAs in Berlin to systematically collect additional information on hepatitis A cases, notified as of calendar week 46/2016, in a specifically designed spreadsheet, including information on sexual contacts, sex in non-household venues and drug use, during their assumed period of infection. SOHSA collated case information submitted electronically by LPHAs.

LPHAs were also asked to organise sequencing of hepatitis A virus (HAV) from IgM positive serum samples or stool samples of cases notified as of calendar week 50 at the National Consultant Laboratory for Hepatitis A and Hepatitis E in Regensburg. Nucleic acid isolation, quantitative reverse transcription PCR (RT-qPCR) and sequencing were conducted as described elsewhere [2]. Sequencing primers were chosen according to the HAVNET unified typing protocol [3]. We queried GenBank for sequences with high similarity using the BLAST algorithms. A rooted maximum likelihood phylogenetic consensus tree for sequences of a 437 nucleotide (nt) long fragment in the VP1/P2A junction region was inferred using MEGA version 7.0.18 software.

In order to obtain information about possibly linked cases in other European Union countries, we communicated the information about the increase of hepatitis A in Berlin together with sequence information via the European Centre for Disease Prevention and Control (ECDC)’s Epidemic Intelligence Information System (EPIS) for food- and waterborne diseases and zoonoses (FWD) and the EPIS for sexually transmitted infections.

## Description of the outbreak

As at 20 January 2017, 38 cases of hepatitis A have been notified in Berlin since 14 November 2016 (calendar week 46). Of these, 37 are male, and one is female (Table).

**Table:** Characteristics of notified hepatitis A cases, Berlin, 14 November 2016–20 January 2017 (n=38)

	MSM	Others^a^	Total
Number	30	8	38
Male patients	30 of 30	7 of 8	37 of 38
Median age (range) in years	31 (24–52)	34 (11–50)	31 (11–52)
Hospitalised	6 of 30	2 of 7^b^	8 of 37^b^
Sexual contacts in non-household venues	12 of 24^b^	NA	NA
Migration background	13 of 25^b^	1 of 4^b^	14 of 29
Drug use	1 of 25^b^	NA	NA

MSM: men who have sex with men; NA: not applicable.

^a^ This category includes one heterosexual patient, one homosexual female patient and six male patients with unknown status.

^b^ Information missing for some patients.

Sexual orientation is known for 32 cases (31 men, one woman); 30 identified themselves as MSM, one as heterosexual and the woman as having sex with women (WSW). Median age of the 30 MSM cases is 31 years (range: 24–52 years); they live in seven of the 12 districts in Berlin, and most of them in Mitte (n = 10). Disease onset of MSM cases ranges over an 11-week period (calendar weeks 43/2016–2/2017, Figure 1), which is incompatible with a common exposure to a point source. Three cases are epidemiologically linked to three other notified cases, supporting the assumption of transmission by interpersonal spread. Six cases have a travel history outside Germany (Spain (n = 2), Austria, Greece, Malta, Taiwan (n = 1 each) during the assumed period of infection, but the majority was apparently infected in Germany (likely in Berlin).

**Figure 1:**
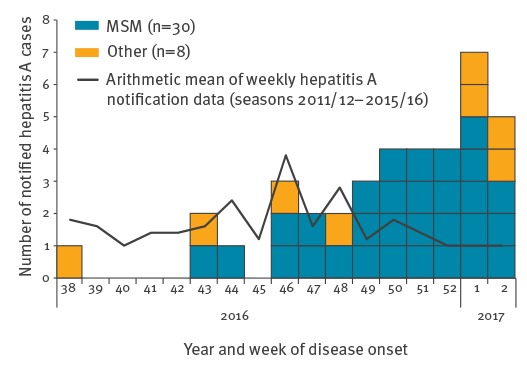
Notified cases of hepatitis A, stratified by sexual orientation and sex by week of symptom onset, Berlin, Germany, 14 November 2016–20 January 2017 (n=38)

None of the MSM cases reported intravenous drug use. One MSM case was vaccinated with one dose of a monovalent hepatitis A vaccine 11 months before disease onset (a second dose within 6 to 12 months after the first dose is usually recommended by manufacturers to provide long-term protection); all others for which information on vaccination is available (n = 27) were unvaccinated (n=23) or their vaccination was incomplete (n=3, single doses of HAV/HBV combination vaccine or unknown vaccine more than one year before disease onset) or outdated (n=1, last dose in 2001).

**Figure 2:**
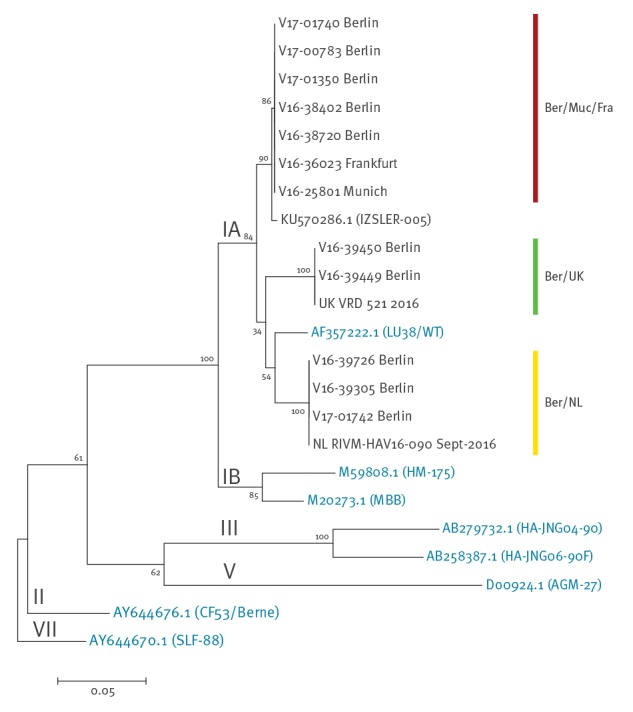
Phylogenetic analysis of hepatitis A viruses, outbreak among men who have sex with men, Berlin, Germany, 14 November 2016–20 January 2017

Sequencing results and phylogenetic analysis show three distinct clusters of MSM-related HAV strains currently circulating in Berlin (Figure 2).

HAV: hepatitis A virus.

Molecular Phylogenetic analysis of the VP1/P2A junction region of selected HAV isolates by Maximum Likelihood method. Genotype VII was used as an outgroup. Sequences are denoted by GenBank ID (reference strains in blue) or isolate ID. Roman numerals indicate genotype; numbers at the nodes indicate bootstrap values.

The evolutionary history was inferred by using the Maximum Likelihood method based on the Tamura-Nei model. All positions containing gaps and missing data were eliminated. There were a total of 437 positions in the final dataset. Sequences from the Netherlands and the United Kingdom are from [4].

The five sequences in the cluster Ber/Muc/Fra (including the WSW) are identical (100% match in the investigated 437 nt long fragment) to the HAV strain first observed in a MSM patient in August 2016 in Munich and later in a MSM patient in Frankfurt (prototype sequence V16–25801). The HAV sequences of three cases in the cluster Ber/NL are identical to the previously reported MSM-related HAV sequence RIVM-HAV16–090, which was isolated from two patients in September 2016, who had visited the EuroPride in Amsterdam in August 2016 [4]. Two of the identified cases fit in the third cluster Ber/UK with also identical sequences as compared with the MSM HAV outbreak strain UK VRD 521 circulating in the United Kingdom (UK) and reported in 2016 [4]. The closest match in the National Center for Biotechnology Information (NCBI) sequence database for the Ber/Muc/Fra cluster was isolated in 2013 in Italy during a multi-country European food-borne outbreak (IZSLER-005, acc. KU570286.1, 99.5% identity) [5], matches for the other clusters are described in [4].

Through EPIS-FWD, colleagues from Austria, Denmark and the Netherlands reported sporadic cases with sequence identity to the Ber/Fra/Muc-Cluster, some of which reported having sex with men in Berlin before disease onset.

## Background

HAV is predominantly transmitted via the faecal-oral route through person-to-person contact or contaminated food and water. The mean incubation period is 28 days (range: 15 to 50 days). Infected persons are most likely to transmit HAV before the onset of jaundice, when HAV concentration in stool is highest [6].Transmission through sexual contact, particularly in MSM [7] as well as through sharing of needles and syringes has also been described [8]. Hepatitis A is a vaccine preventable disease and the German Standing Committee on Vaccination recommends vaccination of people with sexual behaviour at high-risk for HAV infection (such as homosexual contacts) [9]. Recommended vaccinations are paid for by health insurances in Germany.

Germany is a low incidence country with 0.9 notified cases per 100,000 population in 2016. Virtually all HAV infections are directly or indirectly imported [10].

## General and specific public health measures in Germany

In response to the present outbreak, LPHAs educated cases about personal hygiene, traced cases and their contacts and recommended vaccination or post-exposure prophylaxis to contacts according to their risk profile. In addition, LPHA’s, the SOHSA and the Robert Koch Institute (RKI, German national public health institute) formulated prevention recommendations to reinforce offering (i) vaccination to people with sexual behaviour at high-risk for HAV infection [10], and (ii) post-exposure prophylaxis to exposed contacts (active and passive immunisation is effective if administered within two weeks after exposure) [11].

This information was sent to practitioners who focus on treating HIV patients in Berlin, as well as to gay-oriented magazines, newsletters, webpages and specialised healthcare organisations. Furthermore, information was published in the weekly newsletter of the SOHSA and the Epidemiological Bulletin of the RKI [12].

## Discussion

We report on a recent increase of notified hepatitis A cases in Berlin, attributable to cases in MSM. The age distribution of MSM is comparable to that of MSM in previously described hepatitis A outbreaks [7,13]. The vast majority of cases was not vaccinated against hepatitis A indicating a need for targeted risk communication and vaccination campaigns. Of note, condom use is not a safeguard against HAV infection because it does not block the faecal-oral transmission route.

Interestingly, two different HAV sequences detected in cases from Berlin were recently identified in clusters of MSM in the Netherlands and in the UK [7]. The third sequence was identified in a cluster of six MSM cases in Munich and Frankfurt from August through October (data not shown). It is unclear why three different HAV strains concurrently circulate in Berlin among MSM. Apparently, Berlin’s MSM scene is nationally and internationally well connected allowing for ‘importation’ and ‘exportation’ of HAV strains from or to various places in Europe.

For hepatitis A, the German electronic notification system does not capture sexual orientation. Thus, the magnitude of sexually transmitted hepatitis A is likely underestimated. The outbreak described here highlights the interconnectedness of MSM in Europe and the need to increase coverage of hepatitis A vaccination in this group.

## References

[1] Robert Koch Institut (RKI). Falldefinitionen des Robert Koch-Instituts zur Übermittlung von Erkrankungs- oder Todesfällen und Nachweisen von Krankheitserregern, Ausgabe 2015. [Case definitions of the Robert Koch Institute for the surveillance of notifiable infectious diseases in Germany, Edition 2015]. German. Available from: http://www.rki.de/DE/Content/Infekt/IfSG/Falldefinition/Downloads/Falldefinitionen_des_RKI.pdf?__blob=publicationFile

[2] Harries M Monazahian M Wenzel J Jilg W Weber M Ehlers J Foodborne hepatitis A outbreak associated with bakery products in northern Germany, 2012. Euro Surveill. 2014;19(50):20992. 10.2807/1560-7917.ES2014.19.50.20992 25597541

[3] National Institute for Public Health and the Environment (RIVM). Ministry of Health, Welfare and Sport. Protocol. Molecular detection and typing of VP1-2A region of Hepatitis A Virus (HAV). Bilthoven: RIVM. [Accessed 19 Jan 2017]. Available from: http://www.rivm.nl/dsresource?objectid=87925ed7-72d0-4d4f-8b53-fa309fe45b87&type=org&disposition=inline

[4] European Centre for Disease Prevention and Control (ECDC). Hepatitis A outbreaks in the EU/EEA mostly affecting men who have sex with men. Stockholm: ECDC. 19 Dec 2016. Available from: http://ecdc.europa.eu/en/publications/Publications/13-12-2016-RRA-Hepatitis%20A-United%20Kingdom.pdf

[5] Bruni R Taffon S Equestre M Chionne P Madonna E Rizzo C Italian National Task Force on Hepatitis A Key Role of Sequencing to Trace Hepatitis A Viruses Circulating in Italy During a Large Multi-Country European Foodborne Outbreak in 2013. PLoS One. 2016;11(2):e0149642. 10.1371/journal.pone.0149642 26901877 PMC4764681

[6] Mao JS Yu PH Ding ZS Chen NL Huang BZ Xie RY Patterns of shedding of hepatitis A virus antigen in feces and of antibody responses in patients with naturally acquired type A hepatitis. J Infect Dis. 1980;142(5):654-9. 10.1093/infdis/142.5.654 6257794

[7] Cotter SM Sansom S Long T Koch E Kellerman S Smith F Outbreak of hepatitis A among men who have sex with men: implications for hepatitis A vaccination strategies. J Infect Dis. 2003;187(8):1235-40. 10.1086/374057 12696002

[8] Spada E Genovese D Tosti ME Mariano A Cuccuini M Proietti L An outbreak of hepatitis A virus infection with a high case-fatality rate among injecting drug users. J Hepatol. 2005;43(6):958-64. 10.1016/j.jhep.2005.06.012 16143420

[9] Robert Koch Institut (RKI) Statement of the German Standing Committee on Vaccination at the RKI. Recommendations of the Standing Committee on Vaccination. (STIKO) at the Robert Koch Institute/Effective: August 2015. Epidemiol Bull. 2015;332(34). Available from: http://www.rki.de/EN/Content/infections/Vaccination/recommandations/34_2015_engl.pdf?__blob=publicationFile

[10] Faber MS Stark K Behnke SC Schreier E Frank C Epidemiology of hepatitis A virus infections, Germany, 2007-2008. Emerg Infect Dis. 2009;15(11):1760-8. 10.3201/eid1511.090214 19891863 PMC2857222

[11] Victor JC Monto AS Surdina TY Suleimenova SZ Vaughan G Nainan OV Hepatitis A vaccine versus immune globulin for postexposure prophylaxis. N Engl J Med. 2007;357(17):1685-94. 10.1056/NEJMoa070546 17947390

[12] Robert Koch Institut (RKI) Gehäuftes Auftreten von Hepatitis-A-Erkrankungen bei Männern, die Sex mit Männern haben [Increased incidence of hepatitis A among men who have sex with men]. Epidemiol Bull. 2017;2:28 German. Available from: http://www.rki.de/DE/Content/Infekt/EpidBull/Archiv/2017/Ausgaben/02_17.pdf?__blob=publicationFile

[13] Sfetcu O Irvine N Ngui SL Emerson C McCaughey C Donaghy P Hepatitis A outbreak predominantly affecting men who have sex with men in Northern Ireland, October 2008 to July 2009. Euro Surveill. 2011;16(9):19808. 21392487

